# Early-life clinical and hematological profiles: a comparative study of children with and without sickle cell disease in the first three years of life

**DOI:** 10.1007/s00277-025-06479-8

**Published:** 2025-10-09

**Authors:** Siana Nkya, Isihaka Mahawi, Rehema Shungu, Collin Nzunda, Frida Kaywanga, David Solomon, Theogloria Kerrety, Emmanuel Josephat, Heavenlight Christopher, Doreen Ngowi, Julieth Johansen, Florence Urio, Josephine Mgaya, Upendo Masamu, Clara Chamba, Raphael Sangeda, Fadya Hashim, Emmanuela Ambroise, Lulu Chirande, Agnes Jonathan, Emmanuel Balandya, Solomon Ofori Acquah, Julie Makani

**Affiliations:** 1 Department of Biochemistry and Molecular Biology, Muhimbili University of Health and Allied Sciences, Dar es Salaam, Tanzania; 2 Department of Hematology and Blood Transfusion, Muhimbili University of Health and Allied Sciences, Dar es Salaam, Tanzania; 3 Bugando Medical Centre, Catholic University of Health and Allied Sciences, Mwanza, Tanzania; 4 Tanzania Human Genetics Organisation, Dar es Salaam, Tanzania; 5 Sickle Cell Program, Muhimbili University of Health and Allied Sciences, Dar es Salaam, Tanzania; 6 Pediatric department, Mnazi Mmoja Hospital, Zanzibar, Tanzania; 7 University of Ghana, Accra, Ghana; 8 Department of Hematology and Blood Transfusion, Muhimbili University of Health and Allied Sciences, P.O.Box 65001, Dar es Salaam, Tanzania

**Keywords:** Sickle cell disease, Fetal hemoglobin, Clinical profile, Hematological profile

## Abstract

Sickle cell disease (SCD) has been associated with significant morbidity and mortality, particularly in early childhood. Understanding the hematological and clinical outcomes during the early years of life is crucial for guiding interventions to improve care for affected populations. This study investigates the relationship between fetal hemoglobin (HbF) levels, hematological profiles, and clinical outcomes in children with SCD, from birth to three years of age. By comparing children with and without SCD, the study aimed to identify trends in disease progression and highlight the need for early screening and intervention. This longitudinal study recruited and followed children aged 0–3 years. Clinical complaints and hematological parameters were recorded at each visit. Statistical analyses explored associations between HbF levels, hematological parameters, and clinical outcomes in children with SCD. 414 children were recruited, 35.5% with SCD and 64.5% without SCD. Children with SCD experienced a higher frequency of clinical complaints. The hospital admission rate was higher among children with SCD (19.6%) compared to those without (1.7%) (*p* < 0.007), with severe anemia being the leading cause of admission (56.3%). At birth, children with SCD had higher HbF levels (72.87%) that declined over time. The rate of decline correlated with an increased frequency of clinical events in children with SCD. Additionally, children with SCD had lower red cell indices, and higher reticulocyte count as well as white blood cell count. The results emphasize the need for early intervention and continuous monitoring to manage the disease effectively. Further research is recommended to explore the impact of different interventions on improving the quality of life and survival rates in children with SCD.

**Supplementary Information:**

The online version contains supplementary material available at 10.1007/s00277-025-06479-8.

## Introduction

The early years of children affected by SCD are significantly shaped by a unique story of several clinical problems and hematological complexities [1–3]. SCD is an autosomal recessive disorder, whose pathophysiology revolves around sickling of RBCs under hypoxic conditions causing hemolytic anemia, vaso-occlusion, endothelial dysfunction, and significant lifetime morbidity and early mortality [4, 5]. Over 500,000 children are born with the disease annually, disproportionately over 75% of these reside in Sub-Saharan Africa. The disease is also coupled with a high mortality with about 50–90% of children born in Sub-Saharan Africa dying before adulthood [6, 7]. Tanzania ranks fifth globally for SCD birth prevalence, with over 10,000 births annually and a high under-5 mortality rate of over 5000 deaths annually, making the disease a major public health concern [8–10].

The clinical manifestations of the disease are diverse and vary considerably among patients with SCD. Symptoms begin at 3 months as the levels of fetal hemoglobin (HbF) decline and Sickle Hemoglobin (HbS) becomes predominant. The rate of HbF decline varies between individuals; however, it’s not well established how the variation in HbF decline is associated with clinical manifestation later in life [5, 11, 12].

Over the last two decades, substantial progress has been made in understanding the natural history of sickle cell disease from an early age identifying potential predictors of disease severity, and improving the survival of patients. This has been most feasible in places where newborn screening programs are implemented [3]. While these programs are widely implemented in developed countries, they remain limited in many African countries [13, 14]. However, the situation is changing. Countries like Nigeria, Ghana, and Tanzania are making significant strides in prioritizing SCD as part of their National Strategy for Non-Communicable Diseases (NCDs) [8, 15, 16]. In Tanzania, as in other sub-Saharan African countries, efforts to address SCD have led to critical initiatives that provide valuable data on clinical features, mortality rates, and associated complications [17, 18]. Among these are efforts to establish newborn screening (NBS) programs which have enabled the early identification of patients, allowing researchers to study the progression of the disease from a young age and gain a deeper understanding of the factors influencing its clinical complexity and diversity [8, 19–21].

To contribute to these efforts, we designed a study built on ongoing SCD newborn screening and early screening efforts in Tanzania. To obtain broader knowledge, we compared the clinical and hematological profiles of children with and without SCD during their first three years of life. We also sought to correlate the clinical and hematological profiles of children with SCD with the decline of fetal hemoglobin (HbF) to gain a deeper understanding of its influence on disease manifestation. The goal is to contribute to the body of knowledge and enhance HbF-related management strategies, ultimately improving patient outcomes in early childhood.

## Methodology

### Study design

This observational cohort study, conducted from 2019 to 2024, is part of a broader research initiative aimed at exploring the decline of fetal hemoglobin (HbF) and its genetic determinants in the pathophysiology of sickle cell disease (SCD) among Tanzanian children during their first three years of life. The study utilized established platforms, including the newborn screening (NBS) program for SCD, the national immunization program, and Sickle Pan Africa Research Consortium Affiliated clinics (SPARCO) across three regions in Tanzania, to facilitate early detection, recruitment, and follow-up of study participants.

### Study population and sample size

All children under three years were screened for Sickle cell disease. The study population comprised children under three years of age, either screened for SCD through the NBS program at birth and through immunization clinics or enrolled through the SCD clinics.

Based on data indicating that 8 out of every 1,000 children screened are diagnosed with SCD [8], over 25,000 children were screened during the study period, estimating the identification of approximately 150–200 infants with SCD. An equal number of infants without SCD were enrolled as controls for comparison.

### Inclusion and exclusion criteria

The study included all children of the age of 0–3 years, screened for SCD in either NBS or Sickle cell clinics with at least 1 follow-up visit after enrollment. Children diagnosed with other chronic illnesses that could lead to admissions or blood transfusions unrelated to SCD, as well as those with SCD who were receiving disease-modifying drugs like hydroxyurea, were excluded from the study.

### Enrolment and follow-up

Participants were recruited through two main pathways: newborns identified with SCD during routine NBS, and newly diagnosed children at the SPARCO-affiliated SCD clinics.

All participants and their caregivers received education on SCD inheritance and management, including strategies for pain and fever control, and the recognition of severe symptoms such as stroke, acute chest syndrome, and acute splenic sequestration, which would require hospitalization.

Children diagnosed with SCD were enrolled in SPARCO SCD clinics, where they received standard care (SOC), including folic acid and penicillin prophylaxis (125 mg twice daily for children). Education on malaria prevention, emphasizing the use of insecticide-treated nets and prompt treatment, was provided. While malaria chemoprophylaxis and pneumococcal vaccination were recognized public health interventions, they were not part of the SOC during the study. Children and caregivers were also informed about the benefits of hydroxyurea, though children already receiving it were excluded due to its effect on HbF levels.

To facilitate monitoring and data collection, follow-up visits were scheduled in alignment with the national immunization program at key intervals: 6 weeks, 10 weeks, 3 months, 6 months, 9 months, and 18 months, with two additional visits at 24 and 36 months.

### Data collection procedures

Demographic, clinical, and laboratory procedures.

#### Demographic data

At recruitment, demographic information was collected including hospital identification numbers, date of birth, gender, parental tribal affiliations, area of residence, and contact numbers for follow-up.

#### Clinical profiling

Clinical Data were collected using standardized case report forms at enrollment and follow-up visits. Information included the occurrence and frequency of dactylitis, blood transfusions, febrile illnesses, pain episodes, acute chest syndrome, and hospital admissions. Clinical complications were defined based on the Comprehensive Sickle Cell Center in 2010 [22, 23]. At each follow up visit, clinical complications were recorded based on caregiver report of all episodes experienced by the child since the previous visit.

#### Sickle cell diagnosis and hematological profiling

At enrolment, a four-milliliter cord or peripheral blood sample was collected. Two milliliters of the blood sample were used for a rapid test (Hemotype SC) to determine sickle cell status, a full blood count (FBC), and F-cell quantification.

The Full Blood Count (FBC) parameters were analyzed using the Sysmex XT 2000i instrument, following the manufacturer’s procedures [24]. These included hemoglobin (Hb), red blood cell (RBC) count, mean corpuscular volume (MCV), mean corpuscular hemoglobin (MCH), mean corpuscular hemoglobin concentration (MCHC), white cell count (WCC), platelet count (PLT), mean platelet volume (MPV), and reticulocyte count measurements.

F-cell quantification was conducted by flow cytometry using the FACS Canto BD system. Antibodies, including CD71 + PE and Foetal Haemoglobin Monoclonal Antibody (HBF-1) FITC, were utilized to stain the fetal cells (F Cells). Subsequently, flow cytometry analysis was carried out using FlowJo v10.10 software to quantify the F-cell population accurately.

The remaining blood sample was used to prepare a dried blood spot (DBS) for High-Performance Liquid Chromatography (HPLC) analysis to confirm sickle cell status and determine fetal hemoglobin (HbF) levels. HPLC analysis was performed using the Bio-Rad VARIANT II HPLC system at the Haematology Clinic and Research Laboratory HCRL-MUHAS), following the manufacturer’s procedures [25]. HPLC chromatograms displayed distinct peaks for various hemoglobins: Hemoglobin A (HbA), Hemoglobin S (HbS), Hemoglobin A2 (HbA2), and HbF. Normal hemoglobin (AA) showed major peaks for HbA and HbF, while sickle cell trait (AS) exhibited peaks for HbA, HbF, and HbS. Predominant peaks for HbS and HbF characterized sickle cell disease (SS). Quantification of these peaks allowed accurate identification of hemoglobin types. In the context of the study, individuals with HbSS were considered to have sickle cell disease, while individuals with HbAA and HbAS were considered as those without sickle cell disease.

Follow-up visits included the collection of two milliliters of peripheral blood for the same analyses, excluding sickle cell status assessment.

### Ethics

Ethical approval for this study was obtained from the Muhimbili University of Health and Allied Sciences (MUHAS), Ref. No. DA.282/298/01.C. The study was conducted by the Declaration of Helsinki and adhered to ethical principles for medical research involving human subjects. Permission to conduct the study was sought from all relevant authorities at recruitment and follow-up sites. Informed written consent was obtained from the parents or guardians of the participants. All participant information was handled confidentially, and the study incurred no additional financial burden on participants.

### Data management and analysis

Clinical and laboratory data were double-entered to ensure accuracy and stored in a REDCap database hosted according to international data management standards. Data analysis was performed using R (version 4.3.2).

Descriptive statistics summarized the demographic and clinical characteristics of the study population. Means and standard deviations were calculated for continuous variables, while frequencies and percentages were reported for categorical variables and mean fetal hemoglobin (HbF) levels at birth were also assessed.

To evaluate the relationship between age and the occurrence of clinical events, a generalized estimating equation (GEE) analysis was performed. Clinical outcomes such as blood transfusion, dactylitis, episodes of pain, acute chest syndrome, febrile illness, and hospital admissions were examined about age. The results were presented as crude odds ratios (cOR) with 95% confidence intervals (CI) to assess the likelihood of clinical events occurring with each increase in age.

In addition to the clinical analysis, the relationship between sickle cell status (HbAA, HbAS, HbSS) and HbF levels was analyzed using regression models. Sickle cell status and age were considered independent variables.

Furthermore, a comparative analysis was performed between children with and without sickle cell disease (SCD) to assess clinical outcomes. Statistical analyses were conducted to explore differences in the frequency of clinical events, including blood transfusions, respiratory complications, pain episodes, febrile illnesses, dactylitis, and hospital admissions, across various age intervals.

## Results

### Demographic characteristics

414 children were enrolled in the study, 216 males and 194 females. Out of these, 206 (50.2%), 61(14.9%), and 147 (35.9%) were HBAA, HBAS, and HBSS respectively.

### Clinical profile

Children with sickle cell disease (SCD) experienced a progressive need for blood transfusions, increasing from 18.2% at 6 months to 27.3% by 36 months, with the highest frequency of 27.5% observed at 18 months. In contrast, no transfusions were reported among non-SCD children at any age during the study.

Respiratory complications were also more common in children with SCD, occurring between 18 and 24 months, with 7.5% experiencing issues at 18 months and 3.3% at 24 months. In contrast, respiratory complications were rare in children without SCD, with only minor occurrences at 9 and 24 months.

Pain episodes, a defining feature of SCD, sharply increased in frequency over time among SCD children, reaching a peak of 40% at 18 months and remaining high at 36.4% by 36 months. In contrast, no pain episodes were observed in children without SCD. Similarly, febrile illnesses were more frequent in children with SCD, with rates rising from 25% at 18 months to 31.1% at 24 months, and slightly declining to 27.3% at 36 months. For non-SCD children, febrile illness was rare, with only 5.9% affected at 24 months and 1.4% at 9 months.

Dactylitis was most frequent in children with sickle cell disease (SCD), peaking at 18 months, where it affected 12.5% of the cohort. In comparison, dactylitis was almost absent in children without SCD, with only a single case complaint at 9 months. Hospital admissions were also markedly higher in children with SCD, with the highest rate of 32.5% occurring at 18 months, followed by a gradual decline but remaining significant through 36 months. Among children without SCD, hospital admissions were infrequent, ranging from 1.1 to 10% across age intervals, with the highest rate at 36 months. A summary of the overall frequency of clinical events across all children over the study period is provided in Supplementary Table 1.

Over three years, 71 admissions were recorded among all children, with 57 being unique cases. Hospital admissions were more frequent among children with SCD compared to those without SCD. While admissions in non-SCD children were more concentrated in early infancy, SCD children exhibited a broader range of admission ages, with notable peaks at 18 and 24 months. Severe anemia remained the leading cause of admission among children with SCD, whereas pneumonia was the most common cause among non-SCD children other reasons for admission among the two groups are shown in Table 1.

**Table 1:** Description of the reasons for admission and their frequency in children with and without SCD

Reason for admission	All Children (*N* = 71)	With SCD (SS, *N* = 51)	Without SCD (AA + AS, *N* = 20)
Severe Anaemia	40 (56.3%)	36 (70.6%)	4 (20.0%)
Febrile Illness	9 (12.7%)	5 (9.8%)	4 (20.0%)
Severe Pneumonia	6 (8.5%)	1 (2.0%)	5 (25.0%)
Acute Chest Syndrome	2 (2.8%)	2 (3.9%)	0 (0.0%)
Painful Crisis	3 (4.2%)	2 (3.9%)	1 (5.0%)
Acute Watery Diarrhoea	2 (2.8%)	1 (2.0%)	1 (5.0%)
Hypoglycaemia	2 (2.8%)	0 (0.0%)	2 (10.0%)
Infection	1 (1.4%)	0 (0.0%)	1 (5.0%)
Intestinal Obstruction	1 (1.4%)	1 (2.0%)	0 (0.0%)
Neonatal Jaundice	2 (2.8%)	2 (3.9%)	0 (0.0%)
Abdominal Distension	1 (1.4%)	1 (2.0%)	0 (0.0%)
Convulsions	1 (1.4%)	0 (0.0%)	1 (5.0%)
Total	**71 (100%)**	**51 (100%)**	**20 (100%)**

### Clinical profile and age among SCD patients

To assess the relationship between age and the occurrence of clinical events in children with SCD, a generalized estimating equation (GEE) analysis was performed. The results demonstrated a significant increase in the likelihood of experiencing clinical complications with increasing age (Table 2).

**Table 2:** Relationship between time of follow-up (Age of a child) and occurrence of clinical events, *n* = 132. (GEE)

Variable (Outcomes)	cOR	95% CI	*p *– value
Blood transfusion	1.56	1.27–1.90	< 0.001
Dactylitis	2.13	1.45–3.12	< 0.001
Episode of pain	2.25	1.75–2.89	< 0.001
Acute chest syndrome	1.99	1.25–3.18	0.004
Febrile illness	2.11	1.54–2.90	< 0.001
Admission	1.44	1.15–1.81	0.002

Key: cOR: crude Odds Ratio

Reference is absence of respective clinical events (No; category)

**Fig. 1:**
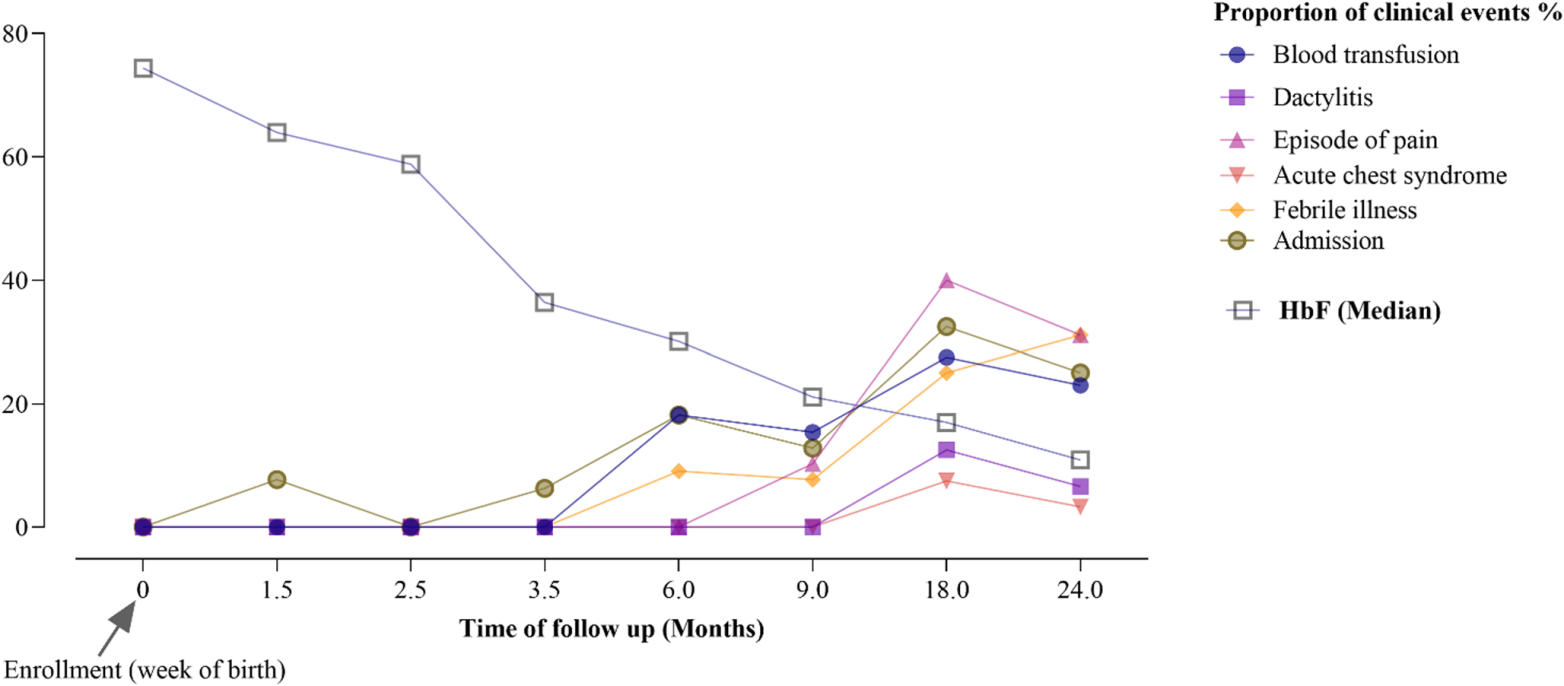
Showing the proportion of clinical events in relation to fetal hemoglobin decline among children with sickle cell disease from birth to 24 months

On average, with each increase in clinical visits, blood transfusion was found to be 1.56 times more likely to occur (cOR = 1.56, 95% CI 1.27–1.90, *p* < 0.001) compared to the previous visit. Additionally, an increase in the average number of visits was associated with the following effects on clinical events: Dactylitis was 2.13 times more likely to occur (cOR = 2.13, 95% CI 1.45–3.12, *p* < 0.001); episodes of pain were 2.25 times more likely (cOR = 2.25, 95% CI 1.75–2.89, *p* < 0.001); acute chest syndrome was 1.99 times more likely (cOR = 1.99, 95% CI 1.25–3.18, *p* = 0.004); febrile illness was 2.11 times more likely (cOR = 2.11, 95% CI 1.54–2.90, *p* < 0.001); and hospital admission was 1.44 times more likely (cOR = 1.44, 95% CI 1.15–1.81, *p* = 0.002) compared to the previous visit. The proportional of clinical events among children with Sickle Cell disease each visit is given in Fig. 1. Overall, the findings demonstrate the significant clinical burden experienced by children with SCD compared to those without the disease, as they grow older.

### Hematological profile of children with and without SCD

When profiling the hematological parameters, we first looked at the rate and pattern of fetal hemoglobin decline. At birth mean HbF levels were higher among children with SCD 72.87(SD 8.5) compared to those without; 65.53 (SD 8.8) and 68.02(SD 7.9) for HbAA and HBAS respectively. In general, we observed a significant decrease in the mean HbF levels as the children aged. However, children with SCD maintained a higher level of HbF (24.34, SD 11.7) compared to children without SCD, whose levels are 11.96 (SD 3.1) and 15.16 (SD 6.9) respectively. Regression analysis results indicated that 44.3% of HbF variance could be explained by sickle status, particularly HbAS, and HbSS, (R² = 0.443, F (3, 653) = 172.890, *p* < 0.001). This model demonstrated that both AS (B = 5.666, *p* < 0.001) and SS (B = 16.865, *p* < 0.001) were significant positive predictors of HbF, indicating higher fetal hemoglobin levels in children with SCD and those who are carriers (HBAS) compared to those without (HBAA). Age (B = −3.060, *p* < 0.001) was a significant negative predictor, confirming that fetal hemoglobin levels decline as age increases. Among children with sickle cell disease, each additional month of age was associated with an average 9.41% decline in HbF levels (β = −9.41, 95% CI −10.56 to −8.26, *p* < 0.001) compared to the previous visit, as illustrated in Fig. 1.

A comparison of full blood counts indicated that individuals with SCD had lower average values for most red cell parameters (RBCs, hemoglobin, MCV, MCH, and MCHC), except for reticulocyte counts (RET) compared to individuals without sickle cell disease. In contrast, children with SCD showed higher WBC counts compared to those without SCD (Table 3). We also analyzed trends in hematological parameters as age increased (Supplementary Fig. 1), observing that WBC counts rose in children with SCD, while RBC counts declined. Reticulocyte counts remained consistently higher in children with SCD. Similar patterns were observed in children with SCD and carriers.

**Table 3:**
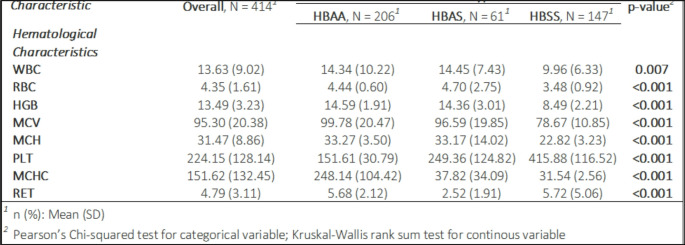
The comparative baseline hematological profiles in children with and without SCD

## Discussion

The early years of life are critical in shaping an individual’s health trajectory into adulthood. This is particularly critical for children with sickle cell disease (SCD). Previous studies have demonstrated that early diagnosis and timely interventions significantly reduce both mortality and morbidity associated with SCD [3]. Therefore, it is essential to understand the early life clinical and hematological profiles of children affected by this condition.

The study was a longitudinal observation cohort aimed to explore the distinct hematological and clinical profiles of children with SCD to highlight the burden faced by children within the first three years of life. The strength of this study lies in the comparison with the non-SCD group which serves to emphasize the disproportionate clinical challenges encountered by children with SCD; however, it is important to note that no group can be considered an ideal comparison for the SCD cohort, given the unique and severe natural history of the disease itself. The study also highlighted the influence of HbF decline on the clinical and hematological profile of children with sickle cell disease.

The results reveal striking differences in clinical outcomes of children with SCD compared to those without. Hospital admissions were significantly more frequent among SCD patients (19.6%) than those without the disease 1.7%. This is critical as it reflects the need for healthcare utilization and proper management of severe clinical manifestations that may require inpatient care. It has been established that in the absence of proper care, only 30–40% of children with SCD survive to their 5th birthday [26, 27]. This also shows the need to enhance well-structured and tailored care plans for children with SCD from an early age to reduce morbidity and mortalities associated with the disease. Recent reports indicate that SCD significantly contributes (7–15%) to under-5 mortality in sub–Saharan African countries [7, 9, 28, 29]. This calls for urgent measures that will address the underlying SCD-associated causes of mortality in a timely and personalized manner.

The findings indicate that children with SCD tend to be admitted to the hospital at older ages and exhibit a more evenly spread age distribution for admissions compared to children without SCD. The higher mean age and broader age range for SCD patients likely reflect the chronic and progressive nature of the disease, necessitating ongoing medical intervention. In contrast, children without SCD are most commonly admitted at a younger age. Other studies have also noted that the rate of hospital admissions for children without chronic illnesses, such as sickle cell disease, tends to decrease after the first year of life. This decline is primarily because infections become less frequent as the immune system matures and becomes more capable of warding off common illnesses [30].

We also observed an increasing occurrence of clinical events with increasing age. This finding aligns with previous studies such as the one conducted in Kilifi, which speculated the protective role of fetal hemoglobin, in early infancy [31–33]. The most common reason for hospital admission in patients with SCD was severe anemia, necessitating an increased need for blood transfusions (17.7% vs. 0.2%, *p* < 0.001). Our findings align with a similar study conducted in Kenya [33]. This shows the importance of considering the need for blood products by children with SCD when implementing national planning. The other common complication for admission for children with SCD included painful episodes (20.3%), febrile illnesses (17.2%), dactylitis, (3.9%), and respiratory complications (2.2%).

Similarly, significant differences were observed in the hematological profiles of children with SCD compared to those without. Children with SCD had lower levels of red blood cell parameters including RBC, HGB, MCV, MCH, and MCHC, but higher RET. Similar findings have been observed in various studies [2, 29, 34]. The observed differences in hematologic parameters have crucial clinical implications. Lower RBC, HGB, MCV, and MCH levels in children with SCD indicate severe anemia and altered red blood cell morphology which are common in SCD. Elevated RET suggests increased red blood cell turnover and compensatory mechanisms for anemia [35]. These hematologic disparities impact the health management of children with SCD, necessitating more frequent monitoring and interventions to manage anemia and prevent complications such as vaso-occlusive crises.

When profiling the hematological parameters, we also studied the rate and pattern of fetal hemoglobin decline and its correlation with SCD. HbF is a known major disease modifier. Rates and patterns of decline of HbF levels differed in children with SCD and those without. Children with SCD had the highest HbF levels at birth 72.87% (SD 8.5) and also maintained almost twice the levels of HbF 24.34% (SD 11.7) at 9 months of age compared to children without SCD (11.96% and 15.16%) for HBAA and HBAS, respectively. This phenomenon also known as delayed switching has been reported in other studies [12, 36]. And has been linked to the protective role against the manifestation of SCD. Supporting this is the correlation of higher levels of HbF with reduced clinical events observed as part of this study and others [11, 12, 33, 37]. Supporting the role of HbF as a potential biomarker for disease severity and response to therapeutic interventions in SCD management. Increased HbF levels tend to reduce HbS polymerization, red blood cell sickling, and hence hemolysis in these patients, thereby decreasing red cell turnover and reducing reticulocyte count [11, 38–41].

While this study represents the first longitudinal study of children with and without SCD in Tanzania, it provides unique insights by studying children from birth and tracking them through immunization clinics and sickle cell clinics. This approach presents both benefits and drawbacks. The active follow-up of patients could have introduced bias, either through over- or under-exaggeration of symptoms associated with self-reporting. Furthermore, because children with SCD were referred to clinics where they received penicillin prophylaxis, folic acid supplementation, and other preventive care, the levels of infections and associated complaints may have been reduced, potentially underestimating the true clinical burden in children with SCD who do not receive such care.While early diagnosis doesn’t prevent complications, It allows timely monitoring and supportive care. While our study doesn’t compare outcomes with settings where interventions are less variable, the observed clinical complications highlight the need for not just screening but also accessible interventions. Without adequate treatment options, early diagnosis may have limited impact, reinforcing the importance of strengthening healthcare systems to ensure early detection accompanied by early management.

The study was also conducted during the COVID-19 pandemic, which impacted clinic attendance and contributed to participant loss over the study duration. Despite these challenges, the study’s strength lies in observing sickle cell disease from an early age, understanding the complexities faced by children, and capturing the diversity of clinical complaints among them. It further highlights how these complications evolve within individuals, offering valuable data to inform interventions and healthcare planning for children with SCD.

However, there is a need for more longitudinal cohort studies that will allow proper monitoring of the disease progression from early childhood through to adulthood. Such studies will provide insights into the long-term effects of early intervention in countries with high burdens of the disease like Tanzania. Additionally, exploring factors that may influence the phenotypic expression of the disease can guide public health interventions in regions with high disease burdens. Such factors include genetic and epigenetics modifiers of disease, as well as environmental factors such as malaria and nutrition status that influence.

## Conclusion

Early-life clinical and hematological profiling is vital for improving SCD outcomes, enabling timely interventions and regular monitoring to prevent complications and enhance the quality of life for affected children. This study underscores significant differences in hematological and clinical profiles among children with and without SCD. More attention should be given to children born with SCD to ensure proper management which will enhance better health outcomes. Elevated fetal hemoglobin (HbF) levels correlate with better clinical outcomes at an early age (0–3 years). This calls for more efforts in developing safe and accessible HbF interventions that can be administered to children born with SCD as soon as they are born or before the age of two years.

## Electronic supplementary material

Below is the link to the electronic supplementary material.

Supplementary Material 1

## Data Availability

Data Availability Statement: The data supporting the findings of this study are available upon reasonable request from the first author, Dr. Siana Nkya, at snkyamtatiro@gmail.com. Access to the data will be granted following appropriate review and approval to ensure ethical and legal compliance.
