# Supplementary material for: Early-life clinical and hematological profiles: a comparative study of children with and without sickle cell disease in the first three years of life
Source: Ann Hematol. 2025 Oct 9;104(10):4949–57. doi: 10.1007/s00277-025-06479-8 (PMC12619737; doi:10.1007/s00277-025-06479-8)
Supplement: Supplementary file 1 — Supplementary Material 1 [file 277_2025_6479_MOESM1_ESM.docx]

**Supplementary Table 1**: Frequencies of Clinical events in the first three years of life.

|  | **With SCD n(%)** | **Without SCD n(%)** |
| --- | --- | --- |
| **Clinical event** |  |  |
| Blood Transfusion | 41(17.7%) | 2(0.2%) |
| Dactylitis | 9(3.9%) | 1(0.1%) |
| Painful episodes | 47(20.3%) | 0(0%) |
| Respiratory Complications | 5(2.2%) | 3(0.3%) |
| Febrile Illness | 40(17.2%) | 3(0.3%) |
| Admission | 45(19.6%) | 17(1.7%) |

**Supplementary Figure 1**: Trends of hematological Parameters with increasing time (age in months) for children with and without SCD.


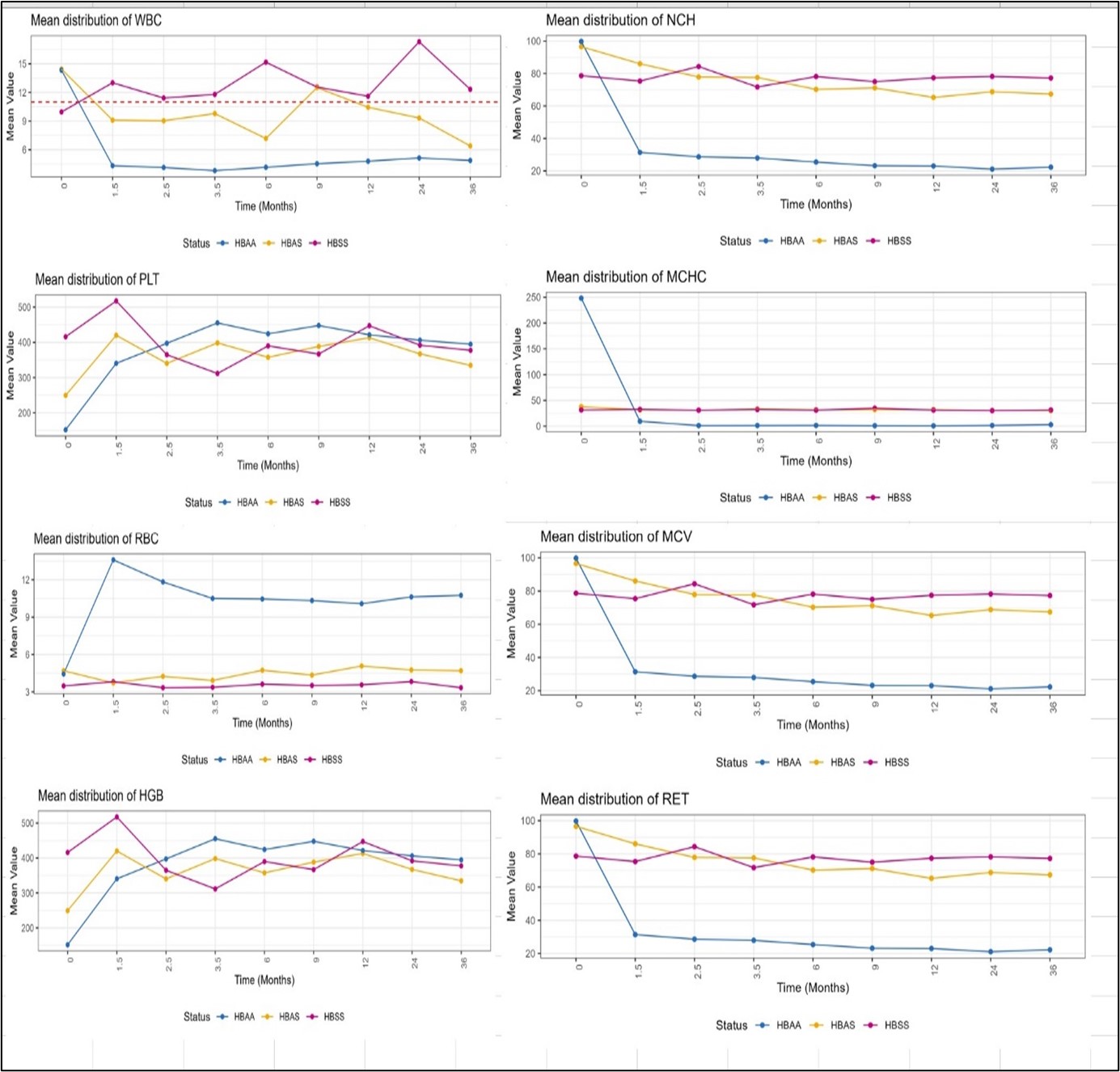


Mean distribution of WBC

Mean distribution of MCH
